# Patient-Derived Induced Pluripotent Stem Cells (iPSCs) and Cerebral Organoids for Drug Screening and Development in Autism Spectrum Disorder: Opportunities and Challenges

**DOI:** 10.3390/pharmaceutics13020280

**Published:** 2021-02-19

**Authors:** Chiara Villa, Romina Combi, Donatella Conconi, Marialuisa Lavitrano

**Affiliations:** School of Medicine and Surgery, University of Milano-Bicocca, 20900 Monza, Italy; romina.combi@unimib.it (R.C.); donatella.conconi@unimib.it (D.C.)

**Keywords:** autism spectrum disorders, induced pluripotent stem cells, brain organoids

## Abstract

Autism spectrum disorder (ASD) represents a group of neurodevelopmental diseases characterized by persistent deficits in social communication, interaction, and repetitive patterns of behaviors, interests, and activities. The etiopathogenesis is multifactorial with complex interactions between genetic and environmental factors. The clinical heterogeneity and complex etiology of this pediatric disorder have limited the development of pharmacological therapies. The major limit to ASD research remains a lack of relevant human disease models which can faithfully recapitulate key features of the human pathology and represent its genetic heterogeneity. Recent advances in induced pluripotent stem cells (iPSCs), reprogrammed from somatic cells of patients into all types of patient-specific neural cells, have provided a promising cellular tool for disease modeling and development of novel drug treatments. The iPSCs technology allowed not only a better investigation of the disease etiopathogenesis but also opened up the potential for personalized therapies and offered new opportunities for drug discovery, pharmacological screening, and toxicity assessment. Moreover, iPSCs can be differentiated and organized into three-dimensional (3D) organoids, providing a model which mimics the complexity of the brain’s architecture and more accurately recapitulates tissue- and organ-level disease pathophysiology. The aims of this review were to describe the current state of the art of the use of human patient-derived iPSCs and brain organoids in modeling ASD and developing novel therapeutic strategies and to discuss the opportunities and major challenges in this rapidly moving field.

## 1. Introduction

Over the past decade, the development of induced pluripotent stem cell (iPSC) technology has provided a novel means to generate disease-relevant cells for in vitro investigation of the molecular and cellular mechanisms underpinning human complex disorders [1,2]. Diseases affecting the central nervous system (CNS) have been the most frequent targets, given the limited access to vital human brain cells and to the lack of or adequacy of existing animal models [3]. iPSCs are undifferentiated cells with self-renewal capability and can be directly generated from somatic cells (isolated from the skin, blood, or urine of an individual) by expressing the reprogramming factors Oct4, Sox2, Klf4, and c-Myc [4]. In principle, the resulting patient-specific iPSCs can subsequently differentiate into disease-relevant cell types, including multiple subclasses of neurons, astrocytes, and microglia, thus providing an unlimited source of cells that harbor the patient’s precise genome associated with the pathogenesis in the appropriate microenvironment. Therefore, the use of iPSCs offers opportunity to study CNS disorders overcoming the ethical limits of human embryonic stem cells (hESCs) or limited durability of primary cultures [5]. Recapitulating disease features, these cells can serve as large-scale drug screening platforms for novel therapeutic targets and have the great potential to be used in cell therapy and personalized medicine as compared to different sources of stem cell, such as hESCs or mesenchymal stem cells (MSCs). Main advantages and disadvantages of these stem cell types are briefly summarized in Table 1.

Although iPSCs-based two-dimensional (2D) brain cultures have increased the understanding of different diseases, human pathologies originate in the context of complex interactions at the cellular, tissue and organ levels. To overcome the inherent constraints associated to iPSCs-derived 2D cultures, three-dimensional (3D) models have been recently implemented, leading to the generation of cerebral organoids, a complex self-organizing 3D aggregate of different cell types derived from iPSCs [6]. Brain organoids possess cerebral-like structures, thus mimicking more closely the in vivo human pathophysiology [7].

Autism spectrum disorder (ASD) encompasses a broad range of complex polygenic and multifactorial neurodevelopmental diseases diagnosed in early childhood (as young as 18 months of age), affecting social interaction, communication, interests, and behavior [8]. The worldwide prevalence of ASD is just under 1% in children with higher estimates in high-income countries and has markedly increased in the last decade probably due to rising awareness of the condition and improved criteria for diagnosis [9]. The core symptoms of ASD vary in degree of severity and are often accompanied by other concomitant diseases and conditions, including epilepsy, anxiety, obsessive compulsive disorder, intellectual disability, schizophrenia (SZ), and attention-deficit hyperactivity disorder (ADHD) [10,11]. Because of the heterogeneity of symptoms and severity of ASD, the disorder may be diagnosed in children at different ages [12]. Most children who are diagnosed with ASD at less than 3 years have retained their diagnosis, while approximately 9% of children who are diagnosed with ASD in early childhood may not meet criteria for ASD by young adulthood [12]. While its exact etiology remains unclear, ASD is known to have a strong genetic background, with family and twins studies suggesting a sizable heritability ranging from 50 to 90% [13,14,15]. The clinical heterogeneity of ASD is due to several factors: the complexity of its genetic profile (the involvement of multiple genes and the existence of different inheritance patterns); the existence of gene-environment interactions; the diverse nature genetic variants involved (ranging from common, rare, de novo and inherited in both coding and non-coding regions of the genome) [16,17]. ASD has been typically classified into non-syndromic (idiopathic, unknown genetic etiology) and syndromic (caused by a known genetic defect), based on a lack of or association to clinical manifestations outside of the autistic features, respectively [18,19]. In the “omics” era, the terms “syndromic” versus “non-syndromic” ASD fare slightly better under careful scrutiny and could be used with appropriate caveats. The use of this classification based on clinical observation demonstrated that it could led to erroneous conclusions that might be corrected retrospectively by means of “omic” studies. For example, rare syndromic mutations were reported in individuals with ASD who were thought not to have syndromic features based on a clinical exam, and, conversely, syndromic features became apparent only by a re-categorization of non-syndromic cohorts after genetic evaluation. Recently, it has become clear that a considerable overlap exists between molecular pathways and biological mechanisms detected in syndromic versus non-syndromic ASD. The term syndromic would be better substituted with “associated with known medical or genetic condition”. Nonetheless, in the description of studies on iPSCs and organoids, we will use the classical classification in syndromic and non-syndromic considering that the cited literature is based on these two groups. In the Supplementary Table S1 resuming the main ASD-associated syndromes, we properly used a classification of syndromic ASD based on the genetic etiology of each disease (monogenic vs. chromosomal disorders; Table S1).

Several autism-susceptibility or genetic risk factors identified in syndromic forms of ASDs involved genes with a role in different processes: the regulation of synaptic plasticity, cell adhesion, neural connectivity, dendritic trafficking, transcription, gene imprinting, chromatin remodeling, and neurotransmission [20,21,22,23,24]. Moreover, modifying risk factors contribute in delineating the observed phenotype. These factors include several pre- and postnatal environmental factors, including maternal smoking, parental age of birth, infection, alcohol consumption, pollution, pesticide exposure, and gestational complications, such as bleeding or diabetes [25,26,27,28,29].

**Figure 1:**
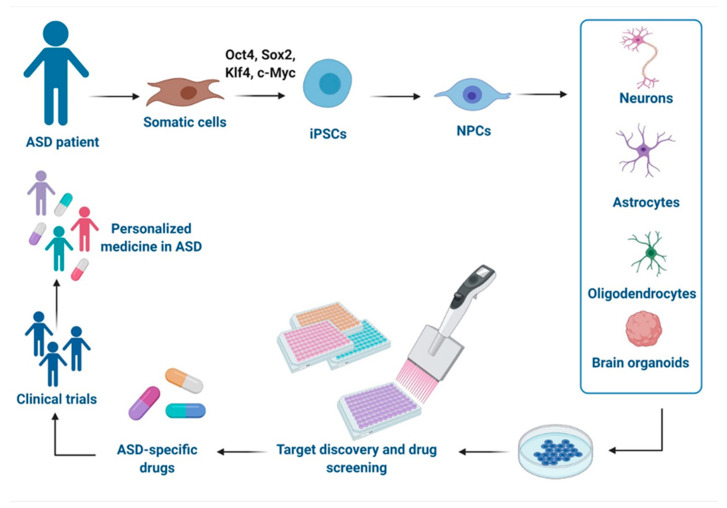
Schematic representation of disease modeling and drug discovery with iPSCs in Autism spectrum disorder (ASD). Specific iPSCs can be derived from somatic cells of ASD individuals and can be further differentiated into different cells types of the central nervous system (CNS) or organized in brain organoids for drug discovery in vitro, development of new drugs, and clinical trials, leading to a personalized medicine for ASD. This figure was created with BioRender.com (accessed on 11 February 2021).

As the multigenic origin of idiopathic ASD makes it difficult to model, iPSCs-based technologies from individuals with genetically complex etiologies have enabled the investigation of pathological mechanisms in disease-relevant human brain cell types and therefore offer a promising platform to test drugs in a safe environment with human genetic background and relevance [30]. Herein, we report advances in modeling ASD with iPSCs-derived technologies and their use in the drug discovery and the development of potential new treatments, as well as their relevance in clinical applications and precision medicine approaches (Figure 1). Moreover, we discuss the challenges of these models.

## 2. iPSC Models of ASDs

The iPSC technology has led to insight into relevant cell types involved in disease mechanisms of ASD, offering a ground-breaking platform to study abnormalities in neurodevelopmental processes that can functionally alter neural circuitry [31]. The majority of iPSCs disease modeling studies use the conventional 2D monolayer culture models to recreate disease-relevant cell types. Two different main strategies are used for producing neural cell starting form iPSCs: the direct differentiation or the reprogramming. In the first case, iPSCs are firstly induced to neural progenitor cells (NPCs) and then differentiated by exposition to trophic factors (e.g., cAMP, BDNF, NT3, and GDNF) [32]. In the reprogramming strategy, iPSCs are transduced with lentiviruses carrying specific transcription factors and an antibiotic selection cassette for efficient conversion and selection [33]. Each strategy shows some advantages and disadvantages compared to the other and their choice is strongly related with the laboratory preference. Different iPSCs-derived phenotypes in NPCs, neurons and glial cells (mainly astrocytes and oligodendrocytes) have been already described for both non-syndromic and syndromic forms of ASD.

### 2.1. iPSCs-Derived Neural Progenitor Cells

NPCs are a population of cells with self-renewal and multipotent differentiation ability that can differentiate into both mature neurons and glial cells. NPCs play an important role in neurogenesis [34], and their dysfunction has been related with both non-syndromic [35,36,37,38] and syndromic ASD, including RETT syndrome (RTT) [39,40,41,42], Fragile X syndrome (FXS) [43,44], tuberous sclerosis complex (TSC) [45,46], Phelan-McDermid syndrome (PMDS) [47], and Timothy syndrome (TS) [48,49].

In regard to idiopathic ASD, NPCs from individuals with macrocephaly displayed an increased cellular proliferation resulted from alterations in a canonical Wnt-β-catenin/BRN transcriptional cascade [35]. These abnormalities in proliferation lead to aberrant neurogenesis and reduced synaptogenesis, thus contributing to functional defects in neuronal networks [35]. Consistently, a recent study demonstrated that the hyperproliferation observed in NPCs derived from iPSCs of ASD subjects with macrocephaly affects genome instability by inducing replication stress-associated genes [36]. Other authors reported that iPSCs-derived NPCs show an impaired crosstalk between the pathways of mTORC1 and Reelin-DAB1, which is known to play a crucial role in regulating neuronal migration and synapse function [37]. This altered interplay was already shown to affect neuronal migration and positioning in a TSC mouse model and in cortical tubers from TSC patients [50]. Moreover, abnormalities in neuronal development, morphology and function were observed in NPCs from a non-syndromic ASD child with a de novo balanced translocation disrupting *TRPC6*, encoding for the human transient receptor potential 6 channel, involved in excitatory synapse and dendritic spine formation [38].

NPCs were also obtained from patients affected by RTT (OMIM #312750), an early-onset neurodevelopmental disorder caused by mutations in the X-linked gene methyl-CpG-binding protein 2 (*MECP2*; Xp28), a ubiquitously expressed transcriptional regulator essential for the maturation and normal function of neurons [51,52]. This syndrome is the second most common cause of severe intellectual disability in females and, a considerable fraction of affected individuals meets diagnostic criteria for ASD [53]. Atypical RTT variants have been identified, associated with mutations in cyclin-dependent kinase-like 5 (*CDKL5*; Xp22.13) and forkhead box protein G1 (*FOXG1*; 14q12) [51]. NPCs derived from iPSCs of patients carrying different *MECP2* mutations underwent X-inactivation when differentiating into functional neurons [39]. Another study found an increase in neural long-interspersed nuclear element-1 (LINE-1) retrotransposon, suggesting that MECP2 is involved in its mobility within the CNS [40]. In three children with *MECP2* duplication, an altered expression of neuronal progenitor genes in NPCs was found [41]. Interestingly, a recent report demonstrated that NPCs and also cortical neurons show a global repressed translation and a decreased ribosome engagement of NEDD4-family ubiquitin ligases, leading to accumulation of target proteins that escape proteasome degradation. These evidences provide insight into novel therapeutic strategies based on the regulation of ubiquination process [42].

FXS (OMIM #300624) is responsible for the most common monogenic cause of ASD that is typically due to a triplet repeat expansion (>200 CGG repeats in the 5′ untranslated region) and subsequent methylation of the *FMR1* gene on the X chromosome (Xq27.3) [54]. As a result, the amount of FMRP (protein product of *FMR1*) decreases, leading to an abnormal translation of different proteins. In fact, FMRP is an mRNA binding protein that regulates the mRNA transport and translational regulation in dendrites [54]. In the case of FXS, Telias et al. performed directed differentiation of cells from three patients and observed in FXS neural progenitors an abnormal expression of key NPC genes (*SOX1*, *NOTCH1*, *PAX6*) [43]. The iPSCs-derived FXS NPCs were then used to investigate functional maturation of the excitatory transmission system in response to the glutamate analog (AMPA) [44]. The authors demonstrated an impairment in calcium (Ca^2+^) signaling via AMPA receptors (AMPARs), suggesting that their functional alterations affect neuronal differentiation and contribute to aberrant neuronal circuit formation and function in FXS [44].

Another ASD-associated syndrome where iPSCs were obtained and studied is TSC (OMIM #191100 and #613254), an autosomal dominant genetic disorder, characterized by benign tumors in the brain and other organs, epilepsy, cognitive impairment, and high penetrance of ASD [55]. The prevalence of ASD in TSC varies among studies, but it is estimated to range from 36% to 50% [55]. TSC is caused by mutations in two genes: *TSC1* (hamartin gene, 9q34) and *TSC2* (tuberin gene, 16p13.3) that act as tumor suppressors inhibiting the mammalian target of rapamycin (mTOR) [56]. These events lead to constitutive activation of mTOR complex 1 cascade and the uncontrolled proliferation of cells. In neurons, dysfunction of this pathway results in abnormal development of fundamental processes that have been proposed to contribute to the behavioral deficits seen in ASD [55]. Human iPSCs-derived from TSC patients exhibited a delay in their ability to differentiate into neurons that is probably related to a dysregulated PI3K/AKT signaling [45]. Recently, isogenic NPCs derived from a patient carrying a nonsense *TSC1* mutation revealed altered early neurodevelopmental phenotypes displaying enhanced proliferation, aberrant neurite outgrowth and enlarged cell size, consistent with mTORC1 activation [46]. However, rapamycin treatment was effective only in reverting enlarged cell size, whereas it was ineffective in increasing proliferation and neurite outgrowth in iPSCs-derived NPCs. Thus, it could be speculated that early neurodevelopmental phenotypes due to *TSC1* loss are not solely related to mTORC1 activation. Moreover, transcriptome analysis showed differentially expressed genes (DEGs) related to ASD phenotype with a genotype-dependent linear response: upregulated/downregulated genes were further increased/decreased in homozygous NPCs, generated by CRISPR/Cas9 (clustered regularly interspaced short palindromic repeats/CRISPR-associated protein 9 nuclease) genome editing technology, compared to the heterozygous state [46].

Similarly, genome-wide RNA sequencing was applied to explore DEGs in iPSCs-NPCs and forebrain neurons derived from PMDS probands and unaffected siblings [47]. PMDS (OMIM #606232), also called 22q13 deletion syndrome, is a rare disease with different clinical features, such as severe intellectual impairments and ASD. A critical region involved three genes, but *SHANK3* is the strongest candidate for the neurobehavioral symptoms [57]. In fact, *SHANK3* encodes a scaffolding protein enriched in the postsynaptic density of glutamatergic synapses and plays a critical role in synaptic function and dendrite formation [58]. In PMDS-iPSCs, the authors found an underexpression of genes involved in Wnt signaling, embryonic development, and protein translation, whereas an overexpression was observed in genes related to pre- and post-synaptic signaling, synaptic plasticity regulation and G-protein-gated potassium channels. Interestingly, these data partially overlapped with iPSC transcriptome findings in other ASDs, converging on altered Wnt signaling and extracellular matrix [47].

In 2011, patient-specific iPSCs-derived NPCs and cortical neurons offered new insights into the pathogenesis of TC (OMIM #601005), a rare and lethal multi-organ disorder connected with gain-of-function missense mutation in the *CACNA1C* gene (12p13.33) [59], linking its phenotype to defects in Ca^2+^ signaling and gene expression-dependent activity [48]. In the same study, the authors reported an abnormal expression of tyrosine hydroxylase (TH) and an increased production of norepinephrine and dopamine in neurons, consistent with the key role of catecholamines in sensory gating and social behavior [48]. A genome-wide weighted co-expression network analysis (WGCNA) on NPCs from TS-iPSCs further confirmed dysregulation of Ca^2+^ signaling in neural development and function [49].

### 2.2. iPSCs-Derived Neurons

Neurons, generated from NSCs during brain development, are electrically excitable cells that transmit information via specialized connections called synapses [60]. Post-mortem human brain analysis, as well as functional and structural imaging studies in ASD, showed an impairment in the formation of neuronal networks and synaptogenesis [61,62]. In addition, cerebral tissues displayed altered neuronal phenotype, consisting of reduced soma size, abnormal neuronal morphology, reduced dendritic arborization, fewer dendritic spines and synapses [63,64]. Similar morphological alterations were also found in iPSCs-derived neurons from non-syndromic ASD patient carrying a *TRPC6* mutation [38]. The same authors demonstrated that the treatment with insulin-like growth factor 1 (IGF-1) or hyperforin, a TRPC6-specific agonist, is able to rescue the neuronal abnormalities, suggesting a possible target for therapeutic strategies in individuals with alterations in this pathway. Interestingly, it has been also shown that MECP2 levels affect *TRPC6* expression, hypothesizing common pathways among ASD [38]. Additional iPSCs-based studies in idiopathic ASD reported an imbalance between excitatory and inhibitory synapses, leading to deficits in social interaction and behaviors as widely described in autistic individuals [65,66,67,68]. An overproduction of GABAergic inhibitory neurons has been observed in iPSCs-derived neurons from patients carrying *FOXG1* mutations [69]. In ASD individuals with macrocephaly, other authors reported a reduced synaptogenesis, abnormal neurogenesis and a significant decrease in both inhibitory and excitatory neurotransmitters, leading to functional defects in neuronal networks which were rescued by IGF-1 treatment [45]. A reduced activity of neurons was further observed in a cohort of non-syndromic ASD patients, showing a significant decrease in excitatory neurotransmitter release, synaptic events and, consequently, spontaneous spike rate [31]. In iPSCs-derived neurons from non-syndromic individuals carrying *SHANK2* mutations, other authors recently showed a hyperconnectivity as demonstrated by an increase in dendrite length and complexity, synapse number and frequency of spontaneous excitatory post-synaptic currents. Moreover, they also reported altered transcriptional level of genes involved in plasticity, synapse and neuronal morphogenesis [70].

Human iPSCs-derived neurons have been largely used to study a wide range of syndromic ASD forms. Concerning RTT, several groups generated iPSC lines from patients and successfully differentiated them into NPCs and functional neurons. Similarly to results reported for NPCs, iPSCs-derived neurons exhibited increased susceptibility for LINE-1 retrotransposition, probably caused by *MECP2* mutations [40]. Marchetto and collaborators [39] showed smaller soma size, reduced dendritic spine densities and fewer synapses in RTT iPSCs-derived neurons in comparison with the isogenic wild-type ones. Moreover, they observed alterations in Ca^2+^ influx, thus causing electrophysiological defects in the RTT neurons [39]. Conversely, other authors reported that cortical neurons, derived from iPSC lines with *MECP2* duplicated gene, display increased synaptogenesis and dendritic complexity with altered neuronal network synchronization recorded by multi-electrode array (MEA) electrophysiology technique [41]. The functional phenotype was further rescued by the treatment with one histone deacetylase inhibitor, named NCH-51 [51]. RNA-seq profiling on differentiated iPSCs-neurons from RTT patients harboring different *MECP2* mutations revealed a prominent enrichment in GABA pathway genes, including GABA receptors and other GABA circuits [71]. Intriguingly, *MECP2*-mutated neurons showed an impairment of the microtubule network along with a significant decrease of acetylated α-tubulin which was reverted by treatment with selective inhibitors of histone deacetylase 6 (HDAC6), the main cytoplasmic deacetylase in which the main substrate is acetylated α-tubulin [71]. In regard to CDKL5 deficiency disorder (CDD), an atypical form of RTT, a study successfully generated clones of *CDKL5*-mutated iPSCs to model disease pathogenesis in vitro [72]. CDD is an X-linked neurological disease caused by pathogenic mutations in the gene for cyclin-dependent kinase-like 5 (*CDKL5*), a serine-threonine kinase highly expressed in the brain [73]. This rare disease was considered as an atypical variant of RTT; however, it has since been recognized as a distinct disorder with common clinical features, such as early-life seizures, autistic behaviors, and intellectual disability [74]. Interestingly, *CDKL5*-mutated iPSCs from females maintained X-chromosome inactivation, allowing the use of clones expressing the wild-type allele as ideal experimental controls, genetically identical to those derived from the same patient [72]. In iPSC-derived neurons from patients carrying *CDKL5* mutations, a following study detected a loss of synaptic contacts, as well as an increased number of aberrant dendritic spines, demonstrating an interesting role for *CDKL5* in spine development and synapse morphogenesis [75].

The majority of studies performed in iPSCs-derived neurons from FXS patients revealed an impairment of neuronal differentiation and maturation as a result of epigenetic differences on *FMR1* gene expression [76,77,78,79,80]. Moreover, neurons exhibited a number of additional phenotypic abnormalities, including an altered electrophysiological network activity, neurite outgrowth and branching defects [80,81]. Perturbations in synaptic transmission, cell proliferation and ion transmembrane transporter activity pathways were also reported [80]. Importantly, other authors demonstrated dysregulated Ca^2+^ signals in iPSCs-derived neurons, strengthening the key role of intracellular Ca^2+^ in neurite growth and synaptic connections [82].

In TSC, iPSCs-derived neurons presented with an enlarged soma, decreased neurite length, complex neurite branching and abnormal connections among cells, as compared to those from unaffected individuals [83]. These abnormalities may be related to hyperactivity of mTOR. Similarly, Zucco et al. generated iPSC-derived NPCs and neurons from two TSC patients reporting a dysregulation of PI3K/AKT/mTORC1 pathway [45]. In mono-cultures of iPSCs-derived cortical neurons, MEA analysis showed an increase in basal dendritic branching and spontaneous Ca^2+^ event frequency [84], consistent with the network hyperactivity previously observed in the TSC mouse model [85]. It should be noted that these aforementioned studies were performed in iPSCs-derived neurons from TSC patients with heterozygous mutations. In a recent study examining iPSCs-derived neurons carrying either single or biallelic mutations in *TSC2*, the authors reported that the loss of one or both alleles of *TSC2* results in mTORC1 hyperactivation and specific neuronal abnormalities which were partially rescued by pharmacological treatment with mTOR regulator rapamycin [86]. However, only neurons harboring biallelic mutations displayed hyperactivity and upregulation of cell adhesion genes observed in cortical tubers. Collectively, these data suggest that the loss of one allele of *TSC2* is sufficient to induce morphological and physiological changes in human neurons, but only with *TSC2* biallelic mutations, a gene expression dysregulation could be detected [86].

In PMDS, neurons derived from iPSCs displayed an impairment in excitatory synaptic transmission, depending on both a failure to generate the correct number of excitatory synapses and a decrease in the expression of glutamate receptors [87]. Interestingly, these defects were rescued by restoring *SHANK3* expression or treating neurons with IGF-1, thus suggesting a key role of *SHANK3* in synapse formation [87]. Moreover, PMDS iPSCs-derived neurons from fibroblasts of 2 children with ASD harboring independent de novo *SHANK3* mutations also provided a novel platform for screening active compounds able to reverse *SHANK3* haploinsufficiency by increasing SHANK3 protein levels and its recruitment to the glutamatergic synapses [88]. The presence of synaptic abnormalities in ASD patients carrying *SHANK3* mutations was further confirmed by a recent study in which iPSCs-derived pyramidal neurons exhibited a significant decrease in dendritic spine densities, as well as in whole spine and spine head volumes [89]. Yi and colleagues studied engineered iPSCs harboring heterozygous and homozygous *SHANK3* deletions reporting significant decreases neurite outgrowth, hyperexcitability, increased input resistance and disrupt excitatory synaptic transmission and demonstrated that these excitability deficits were at least in part due to altered surface expression of hyperpolarization-activated cyclic nucleotide–gated (HCN) channels [90].

As already observed in iPSCs-derived NPCs, also neurons from TS patients showed defects in Ca^2+^ signaling, neuronal differentiation and aberrant expression of TH that was be reversed by treatment with roscovitine, a cyclin dependent kinase inhibitor [48]. Some authors suggested that a neuronal impairment of Ca^2+^ signaling may be caused by the increased intracellular Ca^2+^ influx in rodents and human iPSCs-derived neurons after membrane depolarization. Moreover, they showed that these cells exhibit the activity-dependent dendritic retraction [91]. A recent report suggested how aberrant Ca_V_1.2 splicing affects differentiation of the developing cortex during TS pathogenesis [92].

In the first study using iPSCs-based technology for modeling Angelman syndrome (AS, OMIM #105830) and Prader-Willy Syndrome (PWS, OMIM #176270), no phenotypic differences were observed in neurons [93]. One of the major genes implicated in ASD is *UBE3A* (ubiquitin protein ligase E3A), gene involved in the AS. This disorder is typically caused by a maternal deletion within chromosome 15q11-q13, containing the gene (70 to 75% of cases). Other cases can be ascribed to paternal uniparental disomy (2% to 3%), imprinting center defect (3% to 5%), or single point mutation in the maternal *UBE3A* allele (5 to 10%) [94]. Proper gene dosage of *UBE3A* is crucial to normal brain development, as evidenced by the neurodevelopmental disorders associated with this syndrome [95]. Importantly, the authors found that *UBE3A* imprinting is established during neuronal differentiation of AS iPSCs through an up-regulated expression of paternal *UBE3A* antisense transcript (*UBE3A-ATS*) concomitant with a repression of paternal *UBE3A* [93]. A subsequent study revealed that the silencing of paternal *UBE3A* expression by *SNHG14* (also named as *UBE3A-ATS*) induction is a late event during neuronal differentiation [96]. Moreover, it has been shown that iPSCs-derived neurons from AS individuals harboring a large deletion of 15q11–q13 display impaired maturation of resting membrane potential and action potential firing, a reduction in excitatory synaptic activity and a deficit in activity-dependent synaptic plasticity [97].

### 2.3. iPSCs-Derived Astrocytes

Astrocytes, a sub-type of glia, are the most abundant cells in the mammalian CNS and are involved in homeostasis and defense mechanisms. They also play an active role in neurogenesis, synaptogenesis, neuronal support, neurotransmitter recycling, and neuronal network formation [98]. Although the majority of current applications of iPSCs-based technologies in ASD patients have been focused on neurons, glial cells have been shown to play important role in the disorder. Indeed, post-mortem human brain from children with ASD exhibited abnormal glial cell numbers and function [99], as well as an altered density of excitatory synapses, probably caused by impaired synaptic pruning, the natural process by which extra neurons and synaptic connections are removed in order to increase the neuronal network efficiency [100].

In the case of non-syndromic ASD, only one study has been reported so far focused on the interplay between neurons and astrocytes [31]. Co-culture experiments revealed that astrocytes derived from ASD-iPSCs interfere with proper neuronal development. On the contrary, an improvement in the morphology and synaptogenesis of ASD neurons has been demonstrated by combining them with from control iPSCs-derived astrocytes. The same authors reported a high secretion of the pro-inflammatory cytokine interleukin-6 (IL-6) from astrocytes in ASD individuals probably linked to neural defects and showed an increased synaptogenesis by inhibiting IL-6 [31]. Interestingly, reactive astrocytes have been identified to be the main source of cytokines in post-mortem brain tissue of ASD patients [101] and IL-6 was already reported to be increased in plasma [102,103], cerebrospinal fluid [101] and peripheral blood cells of autistic individuals [104].

Astrocytes derived from iPSCs have been also used to study syndromic ASD, such as RTT. Williams and collaborators specifically focused on the glial effects in RTT neurons by differentiating iPSCs in astroglial progenitors and astrocytes [105]. In co-cultures systems, RTT astrocytes affected the phenotype of hippocampal neurons, causing reduced size of soma and number of terminal ends, as well as shorter neurite length, suggesting that the secretion factors from the astrocytes lead to the aberrant neuronal phenotypes. These morphologic abnormalities were partially rescued when neurons were treated with IGF-1 and GPE (a peptide containing the first 3 amino acids of IGF-1), indicating IGF-1 as a potential cellular target for RTT treatment and highlighting potential avenues for drug development [105]. The critical glial contribution to RTT pathology was further confirmed by another study showing a perturbed astrocyte differentiation in iPSC lines that in turn contributed to the functional immaturity of neurons [106].

### 2.4. iPSCs-Derived Oligodendrocytes

Oligodendrocytes are the myelinating glia of the CNS and originate from oligodendrocyte progenitor cells (OPCs), that hold the capacity to proliferate and migrate. They produce myelin sheath, a greatly extended and modified plasma membrane wrapped around the axons, enabling fast saltatory nerve conduction, axon integrity, and thus contributing to the optimal information processing in complex neural networks [107]. As the demyelination is a hallmark of several diseases, oligodendrocytes have become a great cell source for modeling demyelinating disorders [108]. However, increasing studies suggested a possible role of oligodendrocytes also in the ASD pathogenesis [109]. In the left frontal cortex of autistic patients, a decreased concentration of N-acetyl aspartate (NAA), an amino acid involved in the myelination support, was found [110,111]. Moreover, it has been shown that OPCs isolated from the cortex of TS mice exhibit a more complex morphology and higher protein levels of myelin [112].

Regarding iPSCs-derived oligodendrocytes, only one study has been performed in autistic patients. In the attempt to investigate neuro-glia interactions in TSC phenotypes, the authors found a neuronal hypertrophy and increased axonal density in co-cultures with oligodendrocytes. Moreover, decreased maturation but not cellular proliferation was shown in TSC-oligodendrocytes. Interestingly, pharmacological treatment with rapamycin, mTOR regulator, repressed these defects [84]. Hence, developing models for TSC disease and drug discovery based on the complex interaction between neuronal and glial cells may present a promising tool.

### 2.5. Current Challenges for the Applicability of iPSCs in ASD Modeling

Despite the potential benefits of iPSCs in ASD modeling, there are many challenges that need to be addressed for their applications in clinical setting and pharmacology. The selection of donor cell type (dermal fibroblasts, blood cells or renal tubular cells collected from urine) for the reprogramming process represent the first critical step for the iPSCs generation [4]. It has been shown that skin biopsy-derived dermal fibroblasts and blood cells might carry genetic mutations and chromosomal abnormalities due to exposure to high cell turnover rates and ultra-violet radiation [113]. Depending on the donor cell types, iPSCs can retain some degree of residual epigenetic memory due to an incomplete resetting of the non-CpG methylation patterns during reprogramming processes, thus affecting the differentiation potential [114]. The second step in the generation of iPSCs is the choice of the suitable method for cellular delivery of the reprogramming factors and strategy. The use of retroviruses and lentiviruses can produce an insertional activation of oncogenes and/or inactivation of tumor suppressor genes, leading to a constitutive expression of the reprogramming factors and altering the characteristics and the differentiation potential of iPSCs.

One of the major limitations to the use of iPSCs is the restricted amount of patient-derived cell lines that limits the possibility of quantitative analyses [115]. Another issue arising from iPSCs-based modeling in ASD is represented by the selection of a proper control. Indeed, in the majority of studies, unaffected subjects or family members are used as controls, but this can be questionable since each individual has a unique genetic background that could potentially affect the identification of disease-relevant phenotypes.

Like all cultured cells, iPSCs show natural genetic heterogeneity even in cell lines derived from the same individual. Therefore, such diversity can create difficulties at any step of reprogramming somatic cells to iPSCs and may also impair their differentiation ability into desired cell populations [116].

## 3. Brain Organoid Models of ASDs

Three-dimensional self-organized neural cultures rely on the ability of iPSCs or, more in general, stem cell to self-organize in 3D structures, frequently named mini-brain, providing also required self-patterning factors for morphogenesis and differentiation [117]. These organoids are able to sum up sequential neurogenesis, migration, gliogenesis, and synaptogenesis of cortical development that cannot be studied in 2D cell cultures [118]. Being able to be maintained in vitro for up to two years [119], they could reach later stages of neurodevelopment compared to those that can be studied with 2D cultures and, thus, produce later-born neural cells, including astrocytes and oligodendrocytes [119,120].

One of the first study on brain organoid deriving from cells of patients affected by idiopathic ASD and characterized by macrocephaly [69] reported abnormalities in cell-cycle and synaptic growth. In particular, they noted an increment in the expression of the transcription factor FOXG1 that correlated with an increased production of inhibitory neurons. The study highlights the role of this gene and suggests it as a potential therapeutic target [69]. More recently, Schafer and colleagues [121] performed a large study, based on a selected cohort of macrocephalic ASD patients, in which they combined direct iPSCs-to neuron conversion, transcriptomic analysis and cerebral organoids generation. This strategy made it possible to clarify that ASD-related neurodevelopmental abnormalities are triggered by a specific temporal sequence of gene networks dysregulation. The study showed different results in forebrain organoids than in iPSC-derived neurons. In particular, ASD alterations were well recapitulated in organoids, while the direct conversion of iPSCs to mature neurons abolished ASD-associated phenotypes [121]. Owing to the fact that *CHD8*, a gene encoding for a chromatin-remodeling factor, is mutated in a subgroup of patients with ASD and schizophrenia (SZ), some authors performed an RNA-seq analysis of cerebral organoids derived from iPSCs that are heterozygous for a *CHD8* knockout allele, and from isogenic controls [122]. The study showed that CHD8 regulates the expression of other genes implicated in ASD and SZ, in particular *TCF4* and *AUTS2*, and a large overlap was observed for DEGs found by Mariani et al. [69], especially for genes involved in GABAergic interneuron development. Pathway analysis of DEGs revealed an enrichment of genes involved in regulating Wnt/β-catenin signaling, a druggable pathway [122].

Organoids were used also to study RTT pathogenesis. In particular, Mellios and colleagues [123] analyzed alterations in different neuronal processes, such as neurogenesis, differentiation, and migration, in MECP2-deficient and patient-derived cerebral organoids, while, more recently, Xiang et al. [124] performed a transcriptome analyses revealing a cell-type-specific impairment in specific regions of brain organoids carrying *MECP2* mutations.

In a recent study performed by combining CRISPR/Cas9 genome editing and brain organoids, Blair et al. [125], investigating the “two hit hypothesis” model of cortical tuber formation, demonstrated that homozygous, but not heterozygous, loss of either *TSC1* or *TSC2* impairs the developmental suppression of mTORC1 signaling. Moreover, they demonstrated that the development of TSC phenotype in organoid could be prevented by treating them with rapamycin [125,126].

Brain organoids were also successfully obtained from patient affected by TS in a study that represents the first example of fused organoid approach for the in vitro study neuronal circuits involving different brain regions [127]. In particular, authors studied the migration pattern of inhibitory neurons during brain development using live imaging of TS-forebrain assembloids (fused cerebral organoids resembling the dorsal and ventral forebrain regions). They demonstrated that TS interneurons display a cell-autonomous defect consisting in overall delayed migration from the ventral to the dorsal forebrain [127]. The functional phenotype was rescued by treatment with nimodipine, a drug inhibiting the activity of L-type calcium channel.

Very recently, brain organoids were studied to go deep inside into the pathological mechanisms underlying AS [128]. The study demonstrated that UBE3A suppresses neuronal hyperexcitability via ubiquitin-mediated degradation of calcium and voltage-dependent big potassium (BK) channels. Thus, the use of a BK channels antagonist could normalize neuronal excitability ameliorating also seizure susceptibility [128].

### Current Challenges for the Applicability of Brain Organoids in ASD Modeling

Considering all the above reported studies (summarized in Table 2), besides the high cost of culturing organoids, there are still some challenges facing their use for studying ASD. As compared to 2D monolayer, 3D cultures can be more heterogeneous, displaying high variability in both the structure and the composition of organoids between cell lines, between individual experiments using the same cell lines and even between single organoids derived from the same cell line in the same experiment [129]. The high variability across batches is often the result of the stochastic nature of spontaneous neural in brain organoids, thus making quantitative studies challenging.

A limitation to the use of brain organoids for ASD modeling includes the lack of transport of nutrients and oxygen to cells through the vascularization which can ultimately induce hypoxia and inner necrosis. This also represent an obstacle to the study of blood-brain-barrier (BBB) interactions that is relevant for drug delivery to the brain. Moreover, the lack of vascularization in the neural tissue affects long-term maturation of cerebral organoids, which is extremely relevant to achieve a high degree of cellular complexity and neuronal maturity, including formation of dendritic spines and spontaneously active neuronal networks.

Although brain organoids contain most of the cerebral cell types, microglia are absent from iPSCs-derived brain organoids because of its embryonic origin distinct from that of neural progenitors [130]. As microglia regulate the synaptic pruning, thus contributing to the formation and maintenance of neural circuits [131], it is, therefore, difficult to model these cellular aspects of ASD.

## 4. Use of ASD Models for Drug Discovery and Development

The use of iPSCs as a tool has helped to unveil not only the etiology of ASD, but also aided the progress of the therapeutic areas. Mouse models have routinely been used to test drug candidates, but those identified to date have shown low efficacy in humans, thus limiting their translational potential [132,133]. Moreover, the development of drugs for ASD treatment is still challenging due to the high heterogeneity of the phenotypes, the limited understanding of its pathophysiology, and difficulties in modeling ASD both in vitro and in vivo.

Besides their value for the uncovering of cellular and molecular phenotypes underlying ASD, human iPSCs-based technologies have offered novel possibilities for personalized medicine and for screening drug candidates that can rescue normal phenotype and function. Among them, different iPSCs studies provided evidence that IGF-1 ameliorates some physiological deficits associated with ASD, both in non-syndromic [35,36] and syndromic forms [39,87,105]. In particular, this growth factor was able to restore impaired neuronal network [35], synaptic deficits [38,39,87], or neuronal morphological abnormalities [105] in iPSCs-derived neurons. The evidences coming from iPSCs-based studies in RTT and PMDS modeling have led to a stage II clinical trial on IGF-1 for the treatment of idiopathic and syndromic ASD with results expected in 2022 (https://ClinicalTrials.gov; protocols numbers: NCT01970345; NCT01894958; NCT01777542; NCT01525901; NCT01253317) [134,135]. Furthermore, gentamicin treatment on RTT iPSCs-derived neurons enhanced MECP2 protein levels and increased glutamatergic synapse, further confirming that patient-specific iPSCs can correct neuronal phenotypes [39]. Given *MECP2* function at the epigenetic level, some authors planned to screen a library of 43 compounds with defined activity on epigenetic pathways in order to test the reversibility of the alterations observed in iPSCs-derived neurons harboring *MECP2* duplicated gene. Among them, the histone deacetylase inhibitor NCH-51 was validated as a potential clinical candidate [41]. Intriguingly, in another study performed on iPSCs from *MECP2*-mutated neurons, phenotypic abnormalities were reverted by the treatment with selective inhibitors of HDAC6, the main α-tubulin deacetylase [71]. As mentioned above, other promising results were achieved by roscovitine that can reverse altered expression of TH and increased production of norepinephrine and dopamine displayed in iPSCs-derived neurons from TS individuals carrying a mutation in *CACNA1C* gene [48]. Additionally, the pharmacological intervention with rapamycin, mTOR regulator, successfully recapitulated the increased neuronal activity and morphological changes in TSC iPSCs-derived neurons, suggesting that these cells can also respond to existing drugs in vitro [84,86,125]. In this regard, it should be noted that clinical studies already showed encouraging improvements in TSC patients after treatment with rapamycin [136].

**Figure 2:**
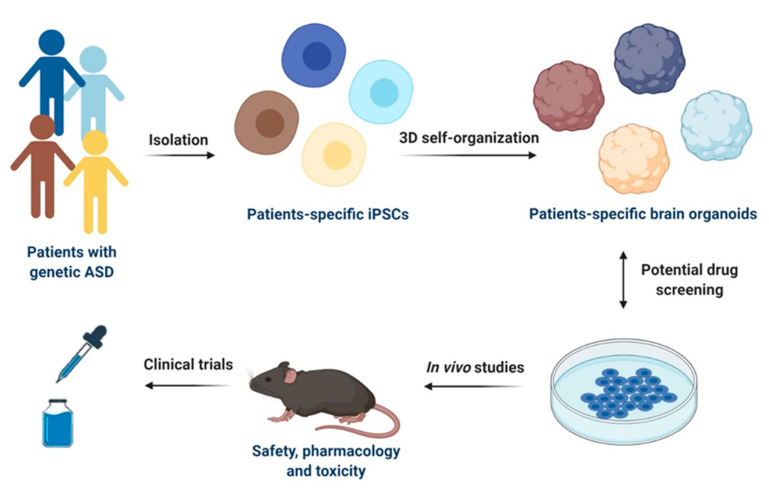
Schematic representation of iPSCs-based models for drug discovery and development in ASD. Specific iPSCs can be isolated from patients with genetic ASD and can be further differentiated into patients-specific iPSCs or 3D self-organized in brain organoids. Potential drug screening can be performed in both 2D cultures of iPSCs-derived cells types of the CNS and in brain organoids. Selected drug candidates can be then firstly tested in in vivo models for safety, pharmacology, and toxicity and then used for clinical trials in humans. This figure was created with BioRender.com (accessed on 11 February 2021).

The aforementioned findings are very promising and suggest that disease-specific models based on iPSCs may represent an emerging and scalable platform for developing novel therapeutic agents targeting molecular mechanisms for ASD treatment (Figure 2). Since iPSCs are renewable, they can provide unlimited cellular resources for large-scale high-throughput screening (HTS). In this regard, one HTS was performed in iPSCs-derived cortical neurons from ASD individuals harboring *SHANK3* mutations to search for compounds that rescue *SHANK3*-associated phenotypes by increasing its mRNA levels. Among 202 tested compounds, lithium and valproic acid were identified as agents with the best efficacy to correct *SHANK3* haploinsufficiency, demonstrating that iPSCs can offer a novel cellular platform for developing specific disease-modifying treatments [88]. However, HTS platforms for drug discovery are still under development and require further standardization, including a homogeneity of cell types and a speeding up of the neuronal maturation by improving the differentiation protocols. Although the current advances in iPSC-based systems hold great promises for drug discovery, these technologies cannot completely replace animal models for preclinical evaluation of drugs.

## 5. Conclusions and Future Directions

ASD is becoming an epidemiologically relevant pediatric disease owing to the increase in the prevalence with time in children [12]. A variety of iPSC-based models have already provided valuable insights into the molecular and cellular mechanisms underpinning several forms of ASD, also offering an unprecedented platform to perform drug screening. However, a number of challenges still remain in using iPSCs for ASD modeling (Table 3). To overcome the limited number of cell lines, one way could consist in the collection of cells derived from both affected and unaffected individuals to be biobanked, thus allowing the large-scale use of iPSCs-based technology. Currently, a biobank has already been created with a collection of about 300 stem cell lines obtained from the tooth pulp of ASD subjects [137] and another with more than 200 fibroblasts, iPSCs, and NSCs, as well as glial cells, generated from ASD individuals and normal unaffected volunteers as controls [138].

The use of genome editing, such as TALEN (transcription activator-like effector nuclease) and CRISPR/Cas9 techniques, is moving to address the selection of a proper control, offering the possibility to generate isogenic cell lines differing only for the specific targeted mutation [139,140]. The use of these technologies can also reduce the problem of line-to-line variability by applying more defined differentiation procedures to both 2D and 3D cultures. Interestingly, 3D bioprinting models have been recently proposed to increase the throughput and reproducibility of organoid generation [141]. This technology can bring together biomaterials, bioactive factors, and cells to build 3D neural tissue, mimicking in vivo neural architecture characteristics and thus allowing the precise control of brain organoid structure [142].

In vivo transplantation of human neural organoids into adult murine brains [143] or the combined use of neural and mesenchymal stem cells [144] should represent valuable solutions for overcoming the lack of transport of nutrients and oxygen. Another approach to incorporate vasculature consists in the supplementation of endothelial vascular growth factor (VEGF) which was shown to successfully promote blood vessel formation in brain organoids [145]. This limitation can also be overcome by the use of engineering devices, such as spinning bioreactors that improve growth conditions, thus allowing the long-term culture of organoids [6]. Miniaturized bioreactors with reduced incubator space and decreased volume of media have also been developed for reducing the high cost of culturing organoids that precludes scalability and compound screening in many laboratories [146].

Despite these current limitations, the combination of iPSCs-based technology with genome editing, organoid engineering or other advances techniques could optimize and refine these iPSCs-derived system models for an extensive clinical application and a better understanding of ASD pathogenesis.

## Figures and Tables

**Table 1:** Main advantages and disadvantages of different types of stem cells: ESCs, MSCs, and iPSCs.

Cell Types	Advantages	Disadvantages
ESCs	Low cost Established protocols for maintenance in culture Any cell type differentiation (pluripotency) Efficient differentiation	Mutation rate Embryo destruction Ethical/political concerns Difficulty to obtain Lack of genetic/immunohistocompatibility match
MSCs	Availability Ease to isolate and expand No ethical concerns Trans-differentiation capacities Success in various clinical applications	Limited number of cell type differentiation (multipotency) Loss of proliferative and differentiation capacities over continuous passages Standardization difficulty Genetic heterogeneity
iPSCs	No ethical concerns Ease to obtain Use of abundant somatic cells of donor Any cell type differentiation (pluripotency) Genetic/immunohistocompatibility match Utility for drug development and developmental studies	Cost of production Difficulty of standardization, reproducibility and maintenance Tumorigenesis Genomic instability

ESCs, embryonic stem cells; MSCs, mesenchymal stem cells; iPSCs: induced pluripotent stem cells.

**Table 2:** Summary of main iPSCs-based studies as a model of ASD.

Disease	Genetic Mutations in Samples (*n*)	iPSCs-Based Models	Relevant Findings	Effective Drugs	Reference
Non-syndromic ASD	different no ASD-related variants (3)	neurons and astrocytes	decreased synapses and release of excitatory neurotransmitters, glial dysfunction, and high levels of IL-6	anti-IL-6	[31]
	different no ASD-related variants (8)	NPCs and neurons	increased proliferation in NPCs, abnormal neurogenesis, reduced synaptogenesis, and decreased release of inhibitory/excitatory neurotransmitters	IGF-1	[35]
	different no ASD-related variants (3)	NPCs	hyperproliferation of NPCs		[36]
	rare compound heterozygous missense variants in *RELN* (1)	NPCs	impaired crosstalk between mTORC1 and Reelin-DAB1 pathways	rapamycin	[37]
	de novo balanced translocation in *TRPC6* (1)	NPCs and neurons	abnormal neuronal development and morphology, fewer dendritic spines and synapses	IGF-1 and hyperforin	[38]
	loss-of-function mutations in *FOXG1* (4)	neurons and brain organoids	overproduction of GABAergic neurons and GABA neurotransmitter		[69]
	heterozygous loss-of-function mutations in *SHANK2* (2)	neurons	increased dendrite length and complexity, synapse number, and frequency of spontaneous excitatory post-synaptic currents	agonist of mGluRs DHPG	[70]
	different no ASD-related variants (8)	brain organoids	neurodevelopmental abnormalities triggered by temporal dysregulation of specific gene networks		[121]
	heterozygous knockout of *CHD8* (1)	brain organoids	enrichment of genes involved in GABAergic interneuron development and Wnt/β-catenin signaling		[122]
RTT	missense, frameshift and nonsense mutations in *MECP2* (4)	NPCs and neurons	reduced soma size, dendritic spine densities and synapses, altered Ca^2+^ signaling, and electrophysiological defects	IGF-1 and gentamicin	[39]
	frameshift mutation in *MECP2* (1)	NPCs and neurons	increased frequency of de novo LINE-1 retrotransposition		[40]
	different duplications in *MECP2* (3)	NPCs and neurons	altered expression of neuronal progenitor genes, increased synaptogenesis and dendritic complexity with altered network synchronization	histone deacetylase inhibitor NCH-51	[41]
	large deletion in *MECP2* (1)	NPCs and cortical neurons	repressed translation and decreased ribosome engagement of NEDD4-family ubiquitin ligases		[42]
	missense mutations in *MECP2* (2)	neurons	impaired microtubule network and decreased acetylated α-tubulin	selective inhibitors of HDAC6	[71]
	missense and nonsense mutations in *MECP2* (3)	neurons and astrocytes	neuronal morphological abnormalities mediated by mutant astrocytes	IGF-1 and GPE	[105]
	missense and nonsense mutations in *MECP2* (2)	astrocytes	perturbed astrocyte differentiation		[106]
	missense and frameshift mutations in *MECP2* (2)	brain organoids	impaired neurogenesis, neuronal differentiation and migration		[123]
	missense and nonsense mutations in *MECP2* (3)	brain organoids	cell-type-specific impairments	BET inhibitor JQ1	[124]
CDKL5 disorder	missense and nonsense mutations in *CDKL5* (2)	neurons	decreased density of dendritic spines and reduced number of excitatory synapse		[72]
	translocation t(7;X) inactivating *CDKL5* (1)	neurons	decreased density of dendritic spines and loss of synaptic contacts		[75]
FXS	>200 CGG repeats in 5′UTR *FMR1* (3)	NPCs	abnormal expression of key NPC genes (*SOX1*, *NOTCH1*, *PAX6*)		[43]
	>200 CGG repeats in 5′UTR *FMR1* (4)	NPCs	impaired Ca^2+^ signaling affecting neuronal differentiation		[44]
	>700 CGG repeats in 5′UTR *FMR1* (3)	neurons	impaired neuronal differentiation and maturation		[76]
	*FMR1* knockout (2)	neurons	abnormal synaptic transmission, neuronal differentiation, and cell proliferation		[80]
	>700 CGG repeats in 5′UTR *FMR1* (3)	neurons	altered neurite outgrowth and branching defects		[81]
	94 CGG repeats in 5′UTR *FMR1* (1)	neurons	dysregulated Ca^2+^ signals, reduced synaptic protein expression, and shorter neurites		[82]
TSC	de novo mutations in *TSC2* (2)	NPCs and neurons	delayed neuronal differentiation		[45]
	nonsense mutation in *TSC1* (1)	NPCs	enhanced proliferation, aberrant neurite outgrowth, and enlarged cell size	rapamycin	[46]
	splicing mutation in *TSC1* (1)	neurons	enlarged soma, decreased neurite length, and abnormal connections		[83]
	de novo mutation in *TSC1* and frameshift mutation in *TSC2* (2)	co-cultures of cortical neurons and oligodendrocytes	cellular hypertrophy and increased axonal density	rapamycin	[84]
	single or biallelic mutations in *TSC2* (2)	neurons	morphological changes	rapamycin	[86]
	loss-of-function mutations in *TSC1* and *TSC2* (2)	brain organoids	impaired developmental suppression of mTORC1 signaling by loss of either *TSC1* or *TSC2*	rapamycin	[125]
PMDS	small/large 22q13.3 deletions and frameshift mutation in *SHANK3* (7)	NPCs	disrupted neurogenesis leading to altered excitatory/inhibitory balance		[47]
	22q13 deletion (2)	neurons	impaired excitatory synaptic transmission	IGF-1	[87]
	de novo truncating and frameshift mutations in *SHANK3* (2)	neurons	impaired excitatory synaptic transmission	lithium and valproic acid	[88]
	de novo truncating mutations in *SHANK3* (4)	pyramidal neurons	decreased dendritic spines and altered spinogenesis		[89]
	heterozygous and homozygous *SHANK3* deletions (2)	neurons	decreased neurite outgrowth, hyperexcitability, increased input resistance, and disrupted excitatory synaptic transmission		[90]
TS	G406R missense mutation in *CACNA1C* (5)	NPCs and neurons	dysregulated Ca^2+^ signaling, impaired neuronal differentiation, increased TH and catecholamine expression	roscovitine	[48]
	G406R missense mutation in *CACNA1C* (3)	NPCs	dysregulated Ca^2+^ signaling affecting neuronal development and function		[49]
	G406R missense mutation in *CACNA1C* (2)	neurons	Ca^2+^-dependent dendritic retraction and altered cellular structure	C3 transferase	[91]
	G406R missense mutation in *CACNA1C* (3)	neurons	altered differentiation in the developing cortex		[92]
	G406R missense mutation in *CACNA1C* (3)	brain organoids	delayed migration of inhibitory neurons	nimodipine	[127]
AS	maternal 15q11-q13 deletion including *UBE3A* (2)	neurons	no phenotypic alterations		[93]
	3bp deletion in the maternally inherited *UBE3A* (1)	neurons	late paternal *UBE3A* silencing during neuronal differentiation		[96]
	large 15q11–q13 deletion (3)	neurons	reduced synaptic activity and plasticity		[97]
	15q11.2-q13 microdeletion including *UBE3A* (1)	brain organoids	neuronal hyperexcitability	BK channels antagonist	[128]
PWS	paternal 15q11-13 deletion (1)	neurons	no phenotypic alterations		[93]

BET, bromodomain and extra-terminal; BK, voltage-dependent big potassium channels; DHPG, 3,5 dihydroxyphenylglycine; GPE, peptide containing the first 3 amino acids of IGF-1; HDAC6, histone deacetylase 6; IGF-1, insulin-like growth factor 1; NPCs, neural progenitor cells; TH, tyrosine hydroxylase.

**Table 3:** Current limitations of iPSCs and potential solutions/approaches in ASD modeling.

Limitations of iPSCs	Potential Solutions/Approaches
Limited amount of patient-derived cell lines	Generation of biobanks of cells derived from patients and unaffected individuals
Lack of proper ASD control	Use of TALEN and CRISPR/Cas9 genome-editing techniques to create isogenic cell lines
Line-to-line variability	Use of TALEN and CRISPR/Cas9 genome-editing techniques to create isogenic cell lines More defined differentiation procedures for both 2D and 3D cultures
Lack of organoid-to-organoid reproducibility	Use of 3D bioprinting models
Lack of vascularization	In Vivo transplantation in animal models Use of combined progenitors (neural and mesenchymal stem cells) Promotion of blood vessel formation by VEGF supplementation in brain organoids
Limited long-term maturation of brain organoids	Optimization of growth conditions by spinning bioreactors In vivo transplantation in animal models
High cost of culturing organoids	Miniaturized bioreactors with reduced incubator space and decreased volume of media

## Data Availability

Not applicable.

## Supplementary Materials

The following are available online at https://www.mdpi.com/1999-4923/13/2/280/s1, Table S1: Main ASD-associated syndromes.
