# Supplementary material for: Patient-Derived Induced Pluripotent Stem Cells (iPSCs) and Cerebral Organoids for Drug Screening and Development in Autism Spectrum Disorder: Opportunities and Challenges
Source: Pharmaceutics. 2021 Feb 19;13(2):280. doi: 10.3390/pharmaceutics13020280 (PMC7922555; doi:10.3390/pharmaceutics13020280)
Supplement: Supplementary file 1 [file pharmaceutics-13-00280-s001.pdf]

pharmaceutics

MDPI

Supplementary Materials: Patient-derived Induced Pluripotent Stem Cells (iPSCs) and Cerebral Organoids for Drug Screening and Development in Autism Spectrum Disorder: Opportunities and Challenges

Chiara Villa [1,*], Romina Combi [1], Donatella Conconi [1] and Marialuisa Lavitrano [1,*]

Table 1. Main ASD-associated syndromes.

Chromosomal Disorders				
**Genetic Bases**	**Disorder (#OMIM)**	**Gene(s)**	**Location**	**iPSC Models [ref]**
Duplication	Chromosome 1q21.1 duplication syndrome (#612475)	Contiguous gene syndromes	1q21.1	n.a.
Deletion	Chromosome 1q21.1 deletion syndrome (#612474)			
Deletion	Chromosome 2q37 deletion syndrome (#600430)	Contiguous gene syndrome	2q37	n.a.
Duplication	Chromosome 7q11.23 duplication syndrome (#609757)	Contiguous gene syndrome	7q11.23	n.a.
Deletion	Williams-Beuren syndrome (#194050)			
Inverted duplication with a terminal deletion	Inverted duplication/deletion 8p21–23 (n.a.)	Multiple genes	8p21–p23	n.a.
Abnormal methylation, paternal uniparental disomy, point mutation of *CDKN1C*, 11p15.5 rearrangement	Beckwith-Wiedemann syndrome (#130650)	*CDKN1C, H19, IGF2, KCNQ1OT1*	11p15.5	n.a.
Duplication	Maternal 15q11–q13 duplication syndrome (#608636)	*UBE3A*	15q11–q13	n.a.
Maternal deletion, paternal uniparental disomy, point mutations of *UBE3A*, imprinting defects	Angelman syndrome (#105830)			[1–4]
Duplication	Paternal 15q11–q13 duplication syndrome (#608636)	*SNRPN, NDN,* possibly others	15q11–q13	n.a.
Paternal deletion, maternal uniparental disomy, imprinting defects	Prader-Willy syndrome (#176270)			[1]
Inverted duplication	Inverted duplicated chromosome 15 syndrome (n.a.)	Multiple genes	15q11.2-q13.1	n.a.
Duplication	Chromosome 16p11.2 duplication syndrome (#614671)	Contiguous gene syndromes	16p11.2	n.a.
Deletion	Chromosome 16p11.2 deletion syndrome (#611913)			
Duplication	Potocki–Lupski syndrome (#610883)	*RAI1,* possibly others	17p11.2	n.a.
Deletion, point mutation of *RAI1*	Smith–Magenis syndrome (#182290)			
Trisomy 21	Down syndrome (#190685)	Multiple genes	chr21	n.a.

Chromosomal Disorders				
**Genetic Bases**	**Disorder (#OMIM)**	**Gene(s)**	**Location**	**iPSC Models [ref]**
Duplication	Chromosome 22q11.21 duplication syndrome (#608363)	Multiple genes	22q11.21	n.a.
Deletion, point mutation of *TBX1*	DiGeorge syndrome (#188400) Velocardiofacial syndrome (#192430)	*TBX1*, possibly others		
Duplication	Chromosome 22q13.3 duplication syndrome (#615538)	*SHANK3*	22q13.3	n.a.
Deletion, point mutation of *SHANK3*	Phelan–McDermid syndrome (#606232)			[5–9]
Monosomy X	Turner syndrome (n.a.)	Multiple genes	chrX	n.a.

Monogenic Disorders				
**Genetic Bases**	**Disorder (#OMIM)**	**Gene(s)**	**Location**	**iPSC Models [ref]**
Point mutation, deletion	Neurexin 1 (*600565)	*NRXN1*	2p16.3	n.a.
Point mutation, deletion	Contactin 4 (*607280)	*CNTN4*	3p26.2–p26.3	n.a.
Point mutation	Protocadherin 10 (*608286)	*PCDH10*	4q28	n.a.
Point mutation	Cornelia de Lange syndrome (#122470)	*NIPBL*	5p13.2	n.a.
Point mutation, deletion	Sotos syndrome (#117550)	*NSD1*	5q35.3	n.a.
Point mutation	Joubert syndrome 3 (#608629)	*AHI1*	6q23.3	n.a.
Point mutation	CHARGE syndrome (#214800)	*CHD7*	8q12.2	n.a.
Point mutation	Cohen syndrome (#216550)	*COH1*	8q22.2	n.a.
Point mutation	Tuberous sclerosis (#191100 and #613254)	*TSC1* *TSC2*	9q34 16p13.3	[10–15]
Point mutation	Macrocephaly/autism syndrome (#605309)	*PTEN*	10q23.31	n.a.
Point mutation	Smith–Lemli–Opitz syndrome (#270400)	*DHCR7*	11q13.4	n.a.
Point mutation	Neurodegeneration due to cerebral folate transport deficiency (#613068)	*FOLR1*	11q13.4	n.a.
Point mutation	Cell adhesion molecule-1 (*605686)	*CADM1*	11q23.3	n.a.
Point mutation	Timothy syndrome (#601005)	*CACNA1C*	12p13.33	[16–20]
Point mutation	Phenylketonuria (#261600)	*PAH*	12q23.2	n.a.
Point mutation	Noonan syndrome (#163950)	*PTPN11*	12q24.13	n.a.
Point mutation	Moebius syndrome (%157900)	Possibly *REV3L* and *PLXND1*	13q12.2-q13	n.a.
Point mutation	FOXG1 deletion syndrome (n.a.)	*FOXG1*	14q12	[21]
Point mutation	Neurofibromatosis type 1 (#162200)	*NF1*	17q11.2	n.a.
Point mutation	Mucopolysaccharidosis type IIIA (#252900)	*SGSH*	17q25.3	n.a.
Point mutation	Helsmoortel–Van der Aa syndrome (#615873)	*ADNP*	20q13.13	n.a.
Point mutation	Adenylosuccinate deficiency (#103050)	*ADSL*	22q13.1	n.a.
Point mutation	Cyclin-dependent kinase-like 5 disorder (n.a.)	*CDKL5*	Xp22.13	[22,23]
Point mutation	Aarskog-Scott syndrome (#305400)	*FGD1*	Xp11.22	n.a.
Point mutation	Lujan–Fryns syndrome (#309520)	*MED12*	Xq13.1	n.a.
Point mutation, deletion	Neuroligin 3 (*300336) Neuroligin 4 (*300427)	*NLGN3* *NLGN4*	Xq13.1 Xp22.32-p22.31	n.a.
Triplet repeat expansion, point mutation	Fragile X syndrome (#300624)	*FMR1*	Xq27.3	[24–29]
Point mutation	Cerebral creatine deficiency syndrome 1 (#300352) Cerebral creatine deficiency syndrome 2 (#612736) Cerebral creatine deficiency syndrome 3 (#612718)	*SLC6A8* *GAMT* *GATM*	Xq28 19p13.3 15q21.1	n.a.
Point mutation	Rett syndrome (#312750)	*MECP2*	Xq28	[30–38]

n.a.: not available.

References

1. Chamberlain, S.J.; Chen, P.F.; Ng, K.Y.; Bourgois-Rocha, F.; Lemtiri-Chlieh, F.; Levine, E.S.; Lalande, M. Induced pluripotent stem cell models of the genomic imprinting disorders Angelman and Prader-Willi syndromes. *Proc. Natl. Acad. Sci. U S A* **2010**, *107*, 17668-17673. doi: 10.1073/pnas.1004487107.
2. Stanurova, J.; Neureiter, A.; Hiber, M.; de Oliveira Kessler, H.; Stolp, K.; Goetzke, R.; Klein, D.; Bankfalvi, A.; Klump, H.; Steenpass, L. Angelman syndrome-derived neurons display late onset of paternal UBE3A silencing. *Sci. Rep.* **2016**, *6*, 30792. doi: 10.1038/srep30792.
3. Fink, J.J.; Robinson, T.M.; Germain, N.D.; Sirois, C.L.; Bolduc, K.A.; Ward, A.J.; Rigo, F.; Chamberlain, S.J.; Levine, E.S. Disrupted neuronal maturation in Angelman syndrome-derived induced pluripotent stem cells. *Nat. Commun.* **2017**, *8*, 15038. doi: 10.1038/ncomms15038.
4. Sun, A.X.; Yuan, Q.; Fukuda, M.; Yu, W.; Yan, H.; Lim, G.G.Y.; Nai, M.H.; D'Agostino, G.A.; Tran, H.D.; Itahana, Y.; *et al*. Potassium channel dysfunction in human neuronal models of Angelman syndrome. *Science* **2019**, *366*, 1486-1492. doi: 10.1126/science.aav5386.
5. Breen, M.S.; Browne, A.; Hoffman, G.E.; Stathopoulos, S.; Brennand, K.; Buxbaum, J.D.; Drapeau, E. Transcriptional signatures of participant-derived neural progenitor cells and neurons implicate altered Wnt signaling in Phelan-McDermid syndrome and autism. *Mol. Autism* **2020**, *11*, 53. doi: 10.1186/s13229-020-00355-0.
6. Shcheglovitov, A.; Shcheglovitova, O.; Yazawa, M.; Portmann, T.; Shu, R.; Sebastiano, V.; Krawisz, A.; Froehlich, W.; Bernstein, J.A.; Hallmayer, J.F.; Dolmetsch, R.E. SHANK3 and IGF1 restore synaptic deficits in neurons from 22q13 deletion syndrome patients. *Nature* **2013**, *503*, 267-271. doi: 10.1038/nature12618.
7. Darville, H.; Poulet, A.; Rodet-Amsellem, F.; Chatrousse, L.; Pernelle, J.; Boissart, C.; Héron, D.; Nava, C.; Perrier, A.; Jarrige, M.; et al. Human Pluripotent Stem Cell-derived Cortical Neurons for High Throughput Medication Screening in Autism: A Proof of Concept Study in SHANK3 Haploinsufficiency Syndrome. *EBioMedicine* **2016**, *9*, 293-305. doi: 10.1016/j.ebiom.2016.05.032.
8. Gouder, L.; Vitrac, A.; Goubran-Botros, H.; Danckaert, A.; Tinevez, J.Y.; André-Leroux, G.; Atanasova, E.; Lemière, N.; Biton, A.; Leblond, C.S.; et al. Altered spinogenesis in iPSC-derived cortical neurons from patients with autism carrying de novo SHANK3 mutations. *Sci. Rep*. **2019**, *9*, 94. doi: 10.1038/s41598-018-36993-x.
9. Yi, F.; Danko, T.; Botelho, S.C.; Patzke, C.; Pak, C.; Wernig, M.; Südhof, T.C. Autism-associated SHANK3 haploinsufficiency causes Ih channelopathy in human neurons. *Science* **2016**, *352*, aaf2669. doi: 10.1126/science.aaf2669.
10. Zucco, A.J.; Pozzo, V.D.; Afinogenova, A.; Hart, R.P.; Devinsky, O.; D'Arcangelo, G. Neural progenitors derived from Tuberous Sclerosis Complex patients exhibit attenuated PI3K/AKT signaling and delayed neuronal differentiation. *Mol. Cell. Neurosci.* **2018**, *92*, 149-163. doi: 10.1016/j.mcn.2018.08.004.
11. Martin, P.; Wagh, V.; Reis, S.A.; Erdin, S.; Beauchamp, R.L.; Shaikh, G.; Talkowski, M.; Thiele, E.; Sheridan, S.D.; Haggarty, S.J.; Ramesh, V. TSC patient-derived isogenic neural progenitor cells reveal altered early neurodevelopmental phenotypes and rapamycin-induced MNK-eIF4E signaling. *Mol. Autism* **2020**, *11*, 2. doi: 10.1186/s13229-019-0311-3.
12. Li, Y.; Cao, J.; Chen, M.; Li, J.; Sun, Y.; Zhang, Y.; Zhu, Y.; Wang, L.; Zhang, C. Abnormal Neural Progenitor Cells Differentiated from Induced Pluripotent Stem Cells Partially Mimicked Development of TSC2 Neurological Abnormalities. *Stem Cell Reports* **2017**, *8*, 883-893. doi: 10.1016/j.stemcr.2017.02.020.
13. Nadadhur, A.G.; Alsaqati, M.; Gasparotto, L.; Cornelissen-Steijger, P.; van Hugte, E.; Dooves, S.; Harwood, A.J.; Heine, V.M. Neuron-Glia Interactions Increase Neuronal Phenotypes in Tuberous Sclerosis Complex Patient iPSC-Derived Models. *Stem Cell Reports* **2019**, *12*, 42-56. doi: 10.1016/j.stemcr.2018.11.019.
14. Winden, K.D.; Sundberg, M.; Yang, C.; Wafa, S.M.A.; Dwyer, S.; Chen, P.F.; Buttermore, E.D.; Sahin, M. Biallelic Mutations in TSC2 Lead to Abnormalities Associated with Cortical Tubers in Human iPSC-Derived Neurons. *J. Neurosci.* **2019**, *39*, 9294-9305. doi: 10.1523/JNEUROSCI.0642-19.2019.
15. Blair, J.D.; Hockemeyer, D.; Bateup, H.S. Genetically engineered human cortical spheroid models of tuberous sclerosis. *Nat. Med*. **2018**, *24*, 1568-1578. doi: 10.1038/s41591-018-0139-y.
16. Paşca, S.P.; Portmann, T.; Voineagu, I.; Yazawa, M.; Shcheglovitov, A.; Paşca, A.M.; Cord, B.; Palmer, T.D.; Chikahisa, S.; et al. Using iPSC-derived neurons to uncover cellular phenotypes associated with Timothy syndrome. *Nat. Med.* **2011**, *17*, 1657-1662. doi: 10.1038/nm.2576.
17. Tian, Y.; Voineagu, I.; Paşca, S.P.; Won, H.; Chandran, V.; Horvath, S.; Dolmetsch, R.E.; Geschwind, D.H. Alteration in basal and depolarization induced transcriptional network in iPSC derived neurons from Timothy syndrome. *Genome Med.* **2014**, *6*, 75. doi: 10.1186/s13073-014-0075-5.
18. Krey, J.F.; Paşca, S.P.; Shcheglovitov, A.; Yazawa, M.; Schwemberger, R.; Rasmusson, R.; Dolmetsch, R.E. Timothy syndrome is associated with activity-dependent dendritic retraction in rodent and human neurons. *Nat. Neurosci*. **2013**, *16*, 201-209. doi: 10.1038/nn.3307.
19. Panagiotakos, G.; Haveles, C.; Arjun, A.; Petrova, R.; Rana, A.; Portmann, T.; Paşca, S.P.; Palmer, T.D.; Dolmetsch, R.E. Aberrant calcium channel splicing drives defects in cortical differentiation in Timothy syndrome. *Elife* **2019**, *8*, e51037. doi: 10.7554/eLife.51037.
20. Birey, F.; Andersen, J.; Makinson, C.D.; Islam, S.; Wei, W.; Huber, N.; Fan, H.C.; Metzler, K.R.C.; Panagiotakos, G.; Thom, N.; *et al*. Assembly of functionally integrated human forebrain spheroids. *Nature* **2017**, *545*, 54-59. doi: 10.1038/nature22330.

21. Mariani, J.; Coppola, G.; Zhang, P.; Abyzov, A.; Provini, L.; Tomasini, L.; Amenduni, M.; Szekely, A.; Palejev, D.; Wilson, M.; et al. FOXG1-Dependent Dysregulation of GABA/Glutamate Neuron Differentiation in Autism Spectrum Disorders. *Cell* **2015**, *162*, 375-390. doi: 10.1016/j.cell.2015.06.034.
22. Amenduni, M.; De Filippis, R.; Cheung, A.Y.; Disciglio, V.; Epistolato, M.C.; Ariani, F.; Mari, F.; Mencarelli, M.A.; Hayek, Y.; Renieri, A.; et al. iPS cells to model CDKL5-related disorders. *Eur. J. Hum. Genet*. **2011**, *19*, 1246-1255. doi: 10.1038/ejhg.2011.131.
23. Ricciardi, S.; Ungaro, F.; Hambrock, M.; Rademacher, N.; Stefanelli, G.; Brambilla, D.; Sessa, A.; Magagnotti, C.; Bachi, A.; Giarda, E.; et al. CDKL5 ensures excitatory synapse stability by reinforcing NGL-1-PSD95 interaction in the postsynaptic compartment and is impaired in patient iPSC-derived neurons. *Nat. Cell. Biol.* **2012**, *14*, 911-923. doi: 10.1038/ncb2566.
24. Telias, M.; Segal, M.; Ben-Yosef, D. Neural differentiation of Fragile X human Embryonic Stem Cells reveals abnormal patterns of development despite successful neurogenesis. *Dev. Biol.* **2013**, *374*, 32-45. doi: 10.1016/j.ydbio.2012.11.031.
25. Achuta, V.S.; Möykkynen, T.; Peteri, U.K.; Turconi, G.; Rivera, C.; Keinänen, K.; Castrén, M.L. Functional changes of AMPA responses in human induced pluripotent stem cell-derived neural progenitors in fragile X syndrome. *Sci. Signal 2018*, *11*, eaan8784. doi: 10.1126/scisignal.aan8784.
26. Sheridan, S.D.; Theriault, K.M.; Reis, S.A.; Zhou, F.; Madison, J.M.; Daheron, L.; Loring, J.F.; Haggarty, S.J. Epigenetic characterization of the FMR1 gene and aberrant neurodevelopment in human induced pluripotent stem cell models of fragile X syndrome. *PLoS One* **2011**, *6*, e26203. doi: 10.1371/journal.pone.0026203.
27. Utami, K.H.; Skotte, N.H.; Colaço, A.R.; Yusof, N.A.B.M.; Sim, B.; Yeo, X.Y.; Bae, H.G.; Garcia-Miralles, M.; Radulescu, C.I.; Chen, Q.; et al. Integrative Analysis Identifies Key Molecular Signatures Underlying Neurodevelopmental Deficits in Fragile X Syndrome. *Biol. Psychiatry* **2020**, *88*, 500-511. doi: 10.1016/j.biopsych.2020.05.005.
28. Doers, M.E.; Musser, M.T.; Nichol, R.; Berndt, E.R.; Baker, M.; Gomez, T.M.; Zhang, S.C.; Abbeduto, L.; Bhattacharyya, A. iPSC-derived forebrain neurons from FXS individuals show defects in initial neurite outgrowth. *Stem Cells Dev*. **2014**, *23*, 1777-1787. doi: 10.1089/scd.2014.0030.
29. Liu, J.; Koscielska, K.A.; Cao, Z.; Hulsizer, S.; Grace, N.; Mitchell, G.; Nacey, C.; Githinji, J.; McGee, J.; Garcia-Arocena, D.; et al. Signaling defects in iPSC-derived fragile X premutation neurons. *Hum. Mol. Genet.* **2012**, *21*, 3795-3805. doi: 10.1093/hmg/dds207.
30. Marchetto, M.C.; Carromeu, C.; Acab, A.; Yu, D.; Yeo, G.W.; Mu, Y.; Chen, G.; Gage, F.H.; Muotri, A.R. A model for neural development and treatment of Rett syndrome using human induced pluripotent stem cells. *Cell* **2010**, *143*, 527-539. doi: 10.1016/j.cell.2010.10.016.
31. Muotri, A.R.; Marchetto, M.C.; Coufal, N.G.; Oefner, R.; Yeo, G.; Nakashima, K.; Gage, F.H. L1 retrotransposition in neurons is modulated by MeCP2. Nature 2010, 468, 443-446. doi: 10.1038/nature09544.
32. Nageshappa, S.; Carromeu, C.; Trujillo, C.A.; Mesci, P.; Espuny-Camacho, I.; Pasciuto, E.; Vanderhaeghen, P.; Verfaillie, C.M.; Raitano, S.; Kumar, A.; et al. Altered neuronal network and rescue in a human MECP2 duplication model. *Mol. Psychiatry* **2016,** *21*, 178-188. doi: 10.1038/mp.2015.128.
33. Rodrigues, D.C.; Mufteev, M.; Weatheritt, R.J.; Djuric, U.; Ha, K.C.H.; Ross, P.J.; Wei, W.; Piekna, A.; Sartori, M.A.; Byres, L.; et al. Shifts in Ribosome Engagement Impact Key Gene Sets in Neurodevelopment and Ubiquitination in Rett Syndrome. *Cell Rep.* **2020**, *30*, 4179-4196.e11. doi: 10.1016/j.celrep.2020.02.107.
34. Landucci, E.; Brindisi, M.; Bianciardi, L.; Catania, L.M.; Daga, S.; Croci, S.; Frullanti, E.; Fallerini, C.; Butini, S.; Brogi, S.; et al. iPSC-derived neurons profiling reveals GABAergic circuit disruption and acetylated α-tubulin defect which improves after iHDAC6 treatment in Rett syndrome. *Exp. Cell. Res.* **2018**, *368*, 225-235. doi: 10.1016/j.yexcr.2018.05.001.
35. Williams, E.C.; Zhong, X.; Mohamed, A.; Li, R.; Liu, Y.; Dong, Q.; Ananiev, G.E.; Mok, J.C.; Lin, B.R.; Lu, J.; et al. Mutant astrocytes differentiated from Rett syndrome patients-specific iPSCs have adverse effects on wild-type neurons. *Hum. Mol. Genet*. **2014**, *23*, 2968-2980. doi: 10.1093/hmg/ddu008.
36. Kim, J.J.; Savas, J.N.; Miller, M.T.; Hu, X.; Carromeu, C.; Lavallée-Adam, M.; Freitas, B.C.G.; Muotri, A.R.; Yates, J.R. 3rd.; Ghosh, A. Proteomic analyses reveal misregulation of LIN28 expression and delayed timing of glial differentiation in human iPS cells with MECP2 loss-of-function. *PLoS One* **2019**, *14*, e0212553. doi: 10.1371/journal.pone.0212553.
37. Xiang, Y.; Tanaka, Y.; Patterson, B.; Hwang, S.M.; Hysolli, E.; Cakir, B.; Kim, K.Y.; Wang, W.; Kang, Y.J.; et al. Dysregulation of BRD4 Function Underlies the Functional Abnormalities of MeCP2 Mutant Neurons. *Mol. Cell.* **2020**, *79*, 84-98. doi: 10.1016/j.molcel.2020.05.016.
38. Blair, J.D.; Hockemeyer, D.; Bateup, H.S. Genetically engineered human cortical spheroid models of tuberous sclerosis. *Nat. Med*. **2018**, *24*, 1568-1578. doi: 10.1038/s41591-018-0139-y.
